# Female *Anopheles gambiae *antennae: increased transcript accumulation of the mosquito-specific odorant-binding-protein OBP2

**DOI:** 10.1186/1756-3305-5-27

**Published:** 2012-02-06

**Authors:** Seth A Hoffman, Lakshminarayanan Aravind, Soundarapandian Velmurugan

**Affiliations:** 1 Sanaria Inc., 9800 Medical Center Dr., Rockville, MD 20850, USA; 2 National Center for Biotechnology Information, National Library of Medicine, National Institutes of Health, Bethesda, MD 20892, USA; 3 Cornell University, 1 Forest Park Lane, Ithaca, NY 14850, USA

**Keywords:** *Anopheles gambiae*, Odorant, Binding, Protein, Antennae

## Abstract

**Background:**

New interventions are required to optimally and sustainably control the *Anopheles *sp. mosquitoes that transmit malaria and filariasis. The mosquito olfactory system is important in host seeking (transmission) and mate finding (reproduction). Understanding olfactory function could lead to development of control strategies based on repelling parasite-carrying mosquitoes or attracting them into a fatal trap.

**Findings:**

Our initial focus is on odorant binding proteins with differential transcript accumulation between female and male mosquitoes. We report that the odorant binding protein, OBP2 (AGAP003306), had increased expression in the antennae of female vs. male *Anopheles gambiae sensu stricto *(G3 strain). The increased expression in antennae of females of this gene by quantitative RT-PCR was 4.2 to 32.3 fold in three independent biological replicates and two technical replicate experiments using *A. gambiae *from two different laboratories. OBP2 is a member of the vast OBP superfamily of insect odorant binding proteins and belongs to the predominantly dipteran clade that includes the *Culex *oviposition kairomone-binding OBP1. Phylogenetic analysis indicates that its orthologs are present across culicid mosquitoes and are likely to play a conserved role in recognizing a molecule that might be critical for female behavior.

**Conclusions:**

OBP2 has increased mRNA transcript accumulation in the antennae of female as compared to male *A. gambiae*. This molecule and related molecules may play an important role in female mosquito feeding and breeding behavior. This finding may be a step toward providing a foundation for understanding mosquito olfactory requirements and developing control strategies based on reducing mosquito feeding and breeding success.

## Findings

### Research hypothesis

Factors that influence mosquito fitness, especially host seeking and mate finding are complex and modulated by multiple cues, of which olfactory cues are most important [1-4]. Detection of odor molecules requires odorant binding proteins (OBPs) that are abundant in antennal chemosensilla [5,6]. OBPs are low molecular weight soluble proteins that bind and transport odor molecules from sensillae to G-protein-coupled receptors in olfactory sensory neurons [6]. The finding of receptor AgamOBP1 binding to its ligand indole demonstrated the significance of OBPs in odor recognition [7]. Understanding olfactory function could lead to development of malaria control strategies based on repelling *Plasmodium *sp. carrying *Anopheles *mosquitoes or attracting them into a fatal trap. A first step is assessment of expression of olfactory system associated genes [7-10]. There is sexually dimorphic expression of OBPs in *Anopheles *mosquitoes and *Drosophila melanogaster *[11-13]. We are focusing on identifying OBPs in antennae of *Anopheles gambiae*, because in Africa *A, gambiae *is the most important vector of *Plasmodium falciparum *[14], a major vector of *Wuchereria bancrofti*, which causes lymphatic filariasis [15], and a vector of O'nyong-nyong virus [16]. In this study, based on results of a screening microarray (unpublished) and previous microarray studies [9,11], we hypothesized that the OBP, OBP2 (AGAP003306), would have increased transcript accumulation by quantitative reverse transcription PCR (qRT-PCR) in female as compared to male *A. gambiae *antennae.

## Methods

### Collection and Processing of RNA

We studied AGAP003306, which had 2 fold greater expression in RNA isolated from antennae of 4 day old *A. gambiae *(Keele strain from Johns Hopkins) females than males in a microarray experiment (unpublished). In another microarray study of RNA from antennae of 5-7 day old Pink-eye *A. gambiae*, expression of AGAP003306 (OBP2) was 1.4 times higher in females as compared to males, but no qRT-PCR was done [9]. In yet another microarray study of RNA isolated from 3 day old whole mosquitoes (Pink-eye strain *A. gambiae*) there was approximately 3 fold increased expression in females vs males [17].

In this study, *A. gambiae sensu stricto *(G3 strain) were from the same batch of eggs (each batch giving rise to a mosquito lot) and were raised to adulthood under standard insectary conditions, and fed *ad libitum *with 10% sugar water [18]. We studied the antennae under controlled conditions of age and exposure to food. Adults of both sexes were collected exactly 4 days after emergence. The mosquitoes were immobilized by exposure to -20°C for 15 minutes, males and females separated, and antennae removed by manual dissection over dry ice, placed into separate 1.5 mL centrifuge tubes and homogenized using a pestle, each in 300 μL of Trizol reagent (Invitrogen, CA). RNA was isolated following manufacturer's instructions and purified using RNeasy mini column (Qiagen). The RNA was then assessed for quality and quantity using NanoDrop (ND-1000). The mosquito antennae that generated the RNA for the qRT-PCR experiments were isolated in July 2009 (mosquitoes from the University of Maryland), and January 2010 and June 2010 (mosquitoes from the National Institutes of Health).

### qRT-PCR Assay

As an endogenous control, and foundation for the qRT-PCR analysis, we used the S7 ribosomal RNA gene of *A. gambiae *[19]. As another control we analyzed AGAP009629, which did not have differential expression in antennae of females vs. males by microarray, but had increased expression in antennae of unfed vs. blood-fed females (unpublished).

The primer pairs synthesized and used (Table 1) were designed using Primer 3[20]. The QuantiTect SYBR Green RT-PCR kit (Qiagen) was used and reactions conducted in 96 well plates for 40 cycles (Applied Biosystems StepOnePlus™ System). Each reaction contained 12.5 μL Qiagen 2x Master Mix, 0.25 μL forward and reverse primers (0.5 μM final concentration), 0.25 μL RT Mix (containing reverse transcriptase), 10.75 μL DEPC water and 1.0 μL of RNA (0.05-1 μg/μL). The 96 well plate was sealed with adhesive film and centrifuged at 3700 rpm for 1 minute at 4°C. The ribosomal S7 gene was used as an endogenous control and a reaction without RT Mix (reverse transcriptase) was included for all reactions as a negative control. Changes in threshold cycles (ΔΔCT) analysis was done to assess the ratio of RNA expression in females vs. males using previously published methods for analysis (Step One Software, v2.2, Applied Biosystems [21,22]).

**Table 1:** Primers used in qRT-PCR

AGAP ID	Forward primer	Reverse primer
AGAP003306	CTGCACATGGGCAAGCTG	CGTTCGCACACATCCTTG
AGAP009629	GCGCTCCCAAGTTCAAAGTA	CGCAATGCACATCGTGTAG
Ribosomal S7	TGCGGCTTCAGATCCGAGTTC	TTCGTTGTGAACCCAAATAAAAATC

### Phylogenetic analysis

Multiple sequence alignments were built using the KALIGN program [23], followed by manual adjustments on the basis of profile-profile and structural alignments. Phylogenetic analysis was conducted using an approximately-maximum-likelihood method implemented in the FastTree 2.1 program under default parameters [24].

## Results

### qRT-PCR

**Figure 1:**
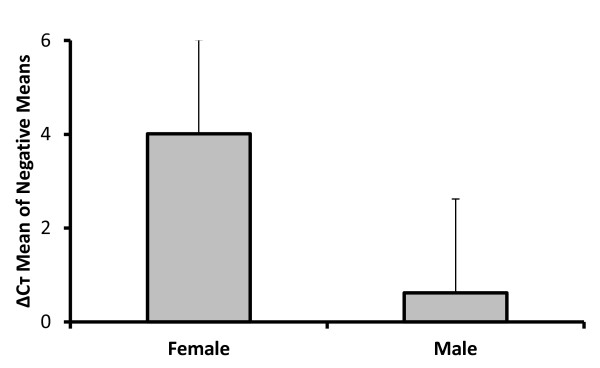
**Comparative expression of AGAP003306 in female and male *A. gambiae***. Columns show mean of ΔCт Mean values (normalized amount of AGAP003306 RNA present) of the three experimental replicates shown in Table 1 multiplied by -1, error bars are standard errors. Data are significantly different in a pair student's t-test (p = 0.037, n = 3).

RNA was extracted from paired antennae of approximately 100 female and 100 male *A. gambiae *derived from the same batch of eggs and raised in the same cage that had never been exposed to a blood meal. 80 ng RNA/mosquito to 560 ng RNA/mosquito (mean of 270 ng RNA/mosquito) was obtained. Approximately double the RNA was obtained from female as compared to male antennae. We isolated antennae on three separate occasions from three separate batches of mosquitoes over a period of 2 years. The results of three assays using RNA isolated from antennae (from three separate lots of mosquitoes) at three different times and normalized to the expression of the S7 ribosomal RNA gene are shown in Table 2. In all experiments there was an increase in accumulation of transcripts of AGAP003306 in female vs. male *A. gambiae *(5 to 25 fold). When these data were integrated (Figure 1), there was a statistically significant increase in transcript accumulation of AGAP003306 in females vs. males (p = 0.037, paired student's t test). Furthermore, there was no significant difference in transcript accumulation between females and males of the control gene, AGAP009629 (p = 0.246, paired student's t test).

**Table 2:** Expression of AGAP003306 (OBP2) and AGAP0099629 (control) relative to expression of S7 in antennae of female and male *A. gambiae *in three biological replicates (experiments 1, 2 and 3).


**Target**	**Experiment**	**Female**			**Male**			**Relative Expression**	
		
		**Cт Mean**	**Cт SD**	**ΔCт Mean**	**Cт Mean**	**Cт SD**	**ΔCт Mean**	**ΔΔCт**	**RQ**

	1	35.55	0.105		36.51	0.257			
**Reference S7**	2	33.31	0.335		35.04	0.125			
	3	33.57	0.565		32.05	0.188			

	1	29.77	0.208	-5.780	33.87	0.414	-2.634	-3.145	8.849
**AGAP003306**	2	29.77	0.168	-3.540	33.87	0.414	-1.170	-2.370	5.171
	3	30.85	0.091	-2.721	33.99	0.309	1.941	-4.662	25.309

	1	35.42	0.298	-0.130	36.06	0.353	-0.450	0.320	0.801
**AGAP009629**	2	35.55	0.105	2.239	36.56	0.390	1.518	0.721	0.607
	3	36.46	0.478	2.894	34.95	0.686	2.894	0.000	1.000

**Cт **= (cycle threshold) number of cycles required for the fluorescent signal to reach threshold

**Cт Mean **= Average Cт from triplicate values on same sample in same PCR reaction

**Cт SD **= Standard Deviation of triplicate Cт

**ΔCт Mean **= Cт MeanTarget- Cт MeanReference (target=AGAP003306 or AGAP009629; reference=S7)

**ΔΔCт **= ΔCт Mean_Female _- ΔCт Mean_Male_

**RQ **= Relative Quantification = 2^-(ΔΔCт)^

The relative increased accumulation of OBP2 transcripts in females vs. males in the three experimental (biological) replicates varied (Table 2). The mosquito antennae that generated the RNA for the qRT-PCR experiments were isolated in July 2009 and were from mosquitoes from the University of Maryland (experiment 1), and in January 2010 (experiment 2) and in June 2010 (experiment 3) both from the National Institutes of Health, Bethesda. Thus, the differences in relative transcript accumulation were likely due to biological variability in gene expression from mosquitoes from different laboratories studied at different times. To determine if this was the case rather than variability of the assay, we repeated the qRT-PCR assays using the RNA of experiments 2 and 3. These technical replicates showed similar results as the original experiments (Table 3). In a recent publication, similar results for the OBP2 gene were found in a single biological replicate of RNA from 4-6 day old *A. gambiae *by RNA-seq [13].

**Table 3:** Expression of AGAP003306 (OBP2) relative to expression of S7 in antennae of female and male A. gambiae in two technical replicates.


**Target**	**Technical replicate**	**Female**			**Male**			**Relative Expression**	
		
		**Cт Mean**	**Cт SD**	**ΔCт Mean**	**Cт Mean**	**Cт SD**	**ΔCт Mean**	**ΔΔCт**	**RQ**

	Experiment 2	33.314	0.335		35.043	0.125			
**Reference**		35.553	0.105		36.507	0.257			
	
**S7**	Experiment 3	33.565	0.565		32.053	0.188			
		36.502	0.309		31.034	0.400			

	Experiment 2	29.774	0.168	-3.540	33.873	0.414	-1.170	-2.371	5.172
**AGAP**		31.725	0.376	-3.828	34.759	0.191	-1.749	-2.079	4.226
	
**003306**	Experiment 3	30.845	0.091	-2.721	33.994	0.309	1.941	-4.662	25.309
		33.167	0.384	-3.335	32.711	0.562	1.678	-5.013	32.293

**Cт **= (cycle threshold) number of cycles required for the fluorescent signal to reach threshold

**Cт Mean **= Average Cт from triplicate values on same sample in same PCR reaction

**Cт SD **= Standard Deviation of triplicate Cт

**ΔCт Mean **= Cт MeanTarget- Cт MeanReference (target=AGAP003306 or AGAP009629; reference=S7)

**ΔΔCт **= ΔCт Mean_Female _- ΔCт Mean_Male_

**RQ **= Relative Quantification = 2^-(ΔΔCт)^

### Phylogenetic Analysis

OBP2 belongs to an OBP super family that includes the insect pheromone binding proteins [6]. Another member of this family, Agam OBP1, mediates indole recognition in antennae of female *A. gambiae *[7]. The olfactory receptors of terrestrial animals exist in an aqueous environment; yet detect odorants that are primarily hydrophobic. The aqueous solubility of hydrophobic odorants is thought to be greatly enhanced via OBPs, which exist in the extracellular fluid surrounding odorant receptors. This family includes proteins that specialize in binding insect pheromones (PBPs) and others that bind general odorants (GOBPs) [6]. Prior phylogenetic analysis has suggested that evolution of the OBP superfamily has evolved primarily through the process of lineage-specific expansion [25]. Thus, the majority of the OBPs in a given lineage such as Diptera, Hymenoptera, Lepidoptera or Coleoptera tend to cluster with others from the same lineage to the exclusion of those from other lineages. The genome of *A. gambiae *itself contains about 72 members of the OBP family.

**Figure 2:**
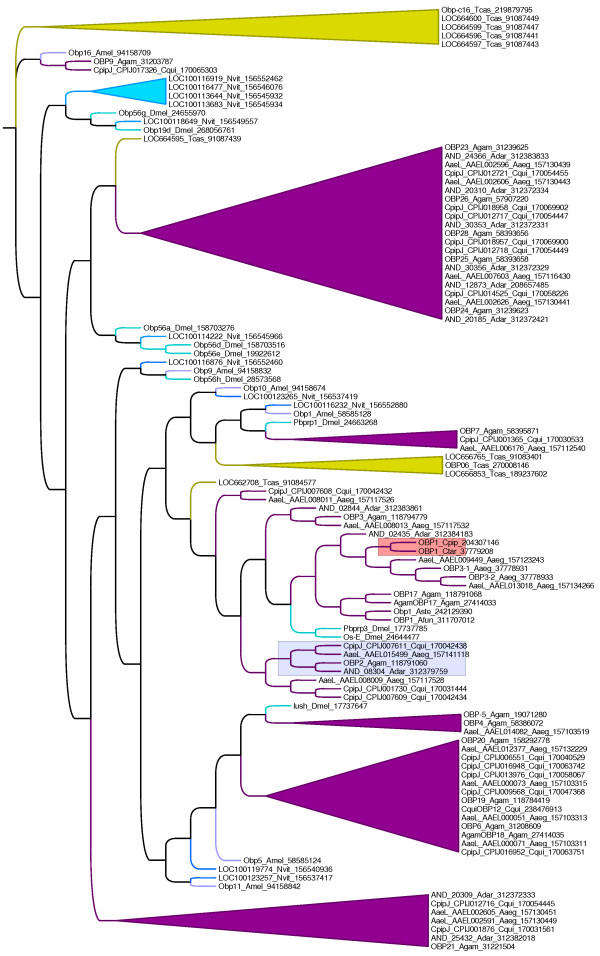
**Phylogenetic analysis**. Phylogenetic analysis was done using over 100 representative OBPs from dipterans (magenta), hymenopterans (turquoise) and coleopterans (yellow). The light blue box shows that orthologs of *A. gambiae *OBP2 are conserved across *Culex, Aedes*, and *Anopheles *genera, but are absent in *Drosophila*. The orange box shows that this clade of OBPs includes the *Culex *(e.g. CquiOBP1) OBP that binds the oviposition kairomones (5R, 6S)-6-acetoxy-5-hexadecanolide. The lineage-specfic expansions in various insect lineages other than the OBP1 and OBP2 clades, which are discussed in the paper have been collapsed and the branch lengths equalized for simplicity of viewing.

We performed a phylogenetic analysis using over 100 representative OBPs from dipterans, hymenopterans and coleopterans with completely sequenced genomes. OBP2 is lodged within a predominantly dipteran lineage-specific expansion of OBPs that are particularly well represented in the culicid mosquitoes (Figure 2). This clade of OBPs includes the *Culex *(e.g. CquiOBP1) OBP that binds the oviposition kairomones (5R, 6S)-6-acetoxy-5-hexadecanolide [26] (marked red, Figure 2). This analysis also showed that orthologs of *A. gambiae *OBP2 are conserved across *Culex, Aedes*, and *Anopheles *genera but are absent in *Drosophila *(blue box, Figure 2), pointing to a function for this protein in potentially binding a conserved odorant molecule in culicid mosquitoes. The up regulation of OBP2 observed in females as compared to males, suggests it could possibly bind a molecule comparable to the oviposition kairomone bound by OBP1. However, such a kairomone could also have an alternative role in guiding female feeding behavior. On the other hand it is also possible that OBP2 binds a pheromone that males express. The focus of future studies would be to pinpoint the role of this protein by determining the impact of knocking it down *vis-a-vis *feeding behavior and fitness of females.

New interventions are needed to control the mosquitoes that transmit the parasites that cause malaria [27,28] and lymphatic filariasis. Despite exciting scientific advances during the past few decades, no new approaches to mosquito vector control have been translated into widely used effective interventions. Sequencing the *A. gambiae *genome [29] and transcriptomics have provided a foundation for an approach to developing new interventions based on identifying genes and gene products that are important in transmission and mate-seeking. Stable genetic knockouts have not been generated in *A. gambiae*. However, transient knockdown by injection of sRNAi can be done and used to confirm the functional importance of OBP2 and other genes. This will be one of the next steps in our work.

## Competing interests

SV is an employee of Sanaria, Inc. Sanaria has no financial interest nor gain, nor competing interest with regard to this manuscript.

## Authors' contributions

SAH: produced the preliminary data on which this work was based, developed the hypothesis and goals for the work, organized mosquito production, and conducted all other aspects of the work. These included dissections of antennae, RNA extractions, qRT-PCR, data analysis, preparation of figures, discussions and manuscript writing; LA: conducted the phylogenetic analysis of the data, prepared figures, and participated in discussions and manuscript writing; SV: was the senior investigator. He established the overall research framework, analysed data, prepared figures, and participated in discussions and manuscript writing. All authors read and approved the final version of the manuscript.

## References

[B1] Zwiebel LJ Takken W Olfactory regulation of mosquito-host interactions Insect Biochem Mol Biol 2004 34 645 652 10.1016/j.ibmb.2004.03.017 15242705 PMC3100215

[B2] Rutzler M Zwiebel LJ Molecular biology of insect olfaction: recent progress and conceptual models J Comp Physiol A Neuroethol Sens Neural Behav Physiol 2005 191 777 790 10.1007/s00359-005-0044-y 16094545

[B3] Chen XG Mathur G James AA Gene expression studies in mosquitoes Adv Genet 2008 64 19 50 19161831 10.1016/S0065-2660(08)00802-X PMC2798853

[B4] Maekawa E Aonuma H Nelson B Yoshimura A Tokunaga F Fukumoto S Kanuka H The role of proboscis of the malaria vector mosquito *Anopheles stephensi *in host-seeking behavior Parasit Vectors 2011 4 10 10.1186/1756-3305-4-10 21272298 PMC3041766

[B5] Vogt RG Riddiford LM Pheromone binding and inactivation by moth antennae Nature 1981 293 161 163 10.1038/293161a0 18074618

[B6] Pelosi P Zhou JJ Ban LP Calvello M Soluble proteins in insect chemical communication Cell Mol Life Sci 2006 63 1658 1676 10.1007/s00018-005-5607-0 16786224 PMC11136032

[B7] Biessmann H Andronopoulou E Biessmann MR Douris V Dimitratos SD Eliopoulos E Guerin PM Iatrou K Justice RW Krober T The *Anopheles gambiae *odorant binding protein 1 (AgamOBP1) mediates indole recognition in the antennae of female mosquitoes PLoS One 2010 5 e9471 10.1371/journal.pone.0009471 20208991 PMC2830424

[B8] Pitts RJ Fox AN Zwiebel LJ A highly conserved candidate chemoreceptor expressed in both olfactory and gustatory tissues in the malaria vector *Anopheles gambiae* Proc Natl Acad Sci USA 2004 101 5058 5063 10.1073/pnas.0308146101 15037749 PMC387373

[B9] Biessmann H Nguyen QK Le D Walter MF Microarray-based survey of a subset of putative olfactory genes in the mosquito *Anopheles gambiae* Insect Mol Biol 2005 14 575 589 10.1111/j.1365-2583.2005.00590.x 16313558

[B10] Xu W Cornel AJ Leal WS Odorant-binding proteins of the malaria mosquito *Anopheles funestus *sensu stricto PLoS One 2010 5 e15403 10.1371/journal.pone.0015403 21042539 PMC2962654

[B11] Schymura D Forstner M Schultze A Krober T Swevers L Iatrou K Krieger J Antennal expression pattern of two olfactory receptors and an odorant binding protein implicated in host odor detection by the malaria vector *Anopheles gambiae* Int J Biol Sci 2010 6 614 626 20975820 10.7150/ijbs.6.614 PMC2962264

[B12] Zhou S Stone EA Mackay TF Anholt RR Plasticity of the chemoreceptor repertoire in *Drosophila melanogaster* PLoS Genet 2009 5 e1000681 10.1371/journal.pgen.1000681 19816562 PMC2750752

[B13] Pitts RJ Rinker DC Jones PL Rokas A Zwiebel LJ Transcriptome profiling of chemosensory appendages in the malaria vector *Anopheles gambiae *reveals tissue- and sex-specific signatures of odor coding BMC Genomics 2011 12 271 10.1186/1471-2164-12-271 21619637 PMC3126782

[B14] Himeidan YE Elzaki MM Kweka EJ Ibrahim M Elhassan IM Pattern of malaria transmission along the Rahad River basin, Eastern Sudan Parasit Vectors 2011 4 109 10.1186/1756-3305-4-109 21679459 PMC3128851

[B15] Dunyo SK Appawu M Nkrumah FK Baffoe-Wilmot A Pedersen EM Simonsen PE Lymphatic filariasis on the coast of Ghana Tran R Soc Trop Med Hyg 1996 90 634 638 10.1016/S0035-9203(96)90414-9 9015499

[B16] Lanciotti RS Ludwig ML Rwaguma EB Lutwama JJ Kram TM Karabatsos N Cropp BC Miller BR Emergence of epidemic O'nyong-nyong fever in Uganda after a 35-year absence: genetic characterization of the virus Virology 1998 252 258 268 10.1006/viro.1998.9437 9875334

[B17] Marinotti O Calvo E Nguyen QK Dissanayake S Ribeiro JM James AA Genome-wide analysis of gene expression in adult *Anopheles gambiae* Insect Mol Biol 2006 15 1 12 10.1111/j.1365-2583.2006.00610.x 16469063

[B18] Benedict MQ Crampton JM, Beard CB, Louis C Care and maintenance of anopheline mosquito colonies The Molecular Biology of Insect Disease Vectors: A Methods Manual 1997 1 London: Chapman and Hall 3 12

[B19] Salazar CE Mills-Hamm D Kumar V Collins FH Sequence of a cDNA from the mosquito *Anopheles gambiae *encoding a homologue of human ribosomal protein S7 Nucleic Acids Res 1993 21 4147 10.1093/nar/21.17.4147 8371989 PMC310024

[B20] Rozen S Skaletsky H Primer3 on the WWW for general users and for biologist programmers Methods Mol Biol 2000 132 365 386 10547847 10.1385/1-59259-192-2:365

[B21] Livak KJ Schmittgen TD Analysis of relative gene expression data using real-time quantitative PCR and the 2(-Delta Delta C(T)) Method Methods 2001 25 402 408 10.1006/meth.2001.1262 11846609

[B22] Schmittgen TD Livak KJ Analyzing real-time PCR data by the comparative C(T) method Nat Protoc 2008 3 1101 1108 10.1038/nprot.2008.73 18546601

[B23] Lassmann T Frings O Sonnhammer EL Kalign2: high-performance multiple alignment of protein and nucleotide sequences allowing external features Nucleic Acids Res 2009 37 858 865 10.1093/nar/gkn1006 19103665 PMC2647288

[B24] Price MN Dehal PS Arkin AP FastTree 2--approximately maximum-likelihood trees for large alignments PLoS One 2010 5 e9490 10.1371/journal.pone.0009490 20224823 PMC2835736

[B25] Lespinet O Wolf YI Koonin EV Aravind L The role of lineage-specific gene family expansion in the evolution of eukaryotes Genome Res 2002 12 1048 1059 10.1101/gr.174302 12097341 PMC186617

[B26] Mao Y Xu X Xu W Ishida Y Leal WS Ames JB Clardy J Crystal and solution structures of an odorant-binding protein from the southern house mosquito complexed with an oviposition pheromone Proc Natl Acad Sci USA 2010 107 19102 19107 10.1073/pnas.1012274107 20956299 PMC2973904

[B27] Breman JG Alilio MS Mills A Conquering the intolerable burden of malaria: what's new, what's needed: a summary Am J Trop Med Hyg 2004 71 1 15 15331814

[B28] WHO The global malaria action plan for a malaria free world. Geneva: Roll Back Malaria Partnership (RBMP) WHO 2008 274

[B29] Holt RA Subramanian GM Halpern A Sutton GG Charlab R Nusskern DR Wincker P Clark AG Ribeiro JM Wides R The genome sequence of the malaria mosquito *Anopheles gambiae* Science 2002 298 129 149 10.1126/science.1076181 12364791

